# Nanoemulsion-enhanced cytotoxicity of *Cyperus scariosus* essential oil in bladder and lung cancer cells: modulation of apoptosis, antioxidant, and cell cycle-related genes

**DOI:** 10.3389/fonc.2026.1939045

**Published:** 2026-09-24

**Authors:** Bruna Silveira Pacheco, Rafaely Piccioni Rosado, Fernanda Severo Sabedra Sousa, Suzana Lemke Lanius, Fabiana Kommling Seixas, Cláudio Martin Pereira de Pereira, Amanda Fonseca Leitzke, Letícia Zibetti, Clarissa Piccinin Frizzo, Janice Luehring Giongo, Rodrigo de Almeida Vaucher, Tiago Veiras Collares

**Affiliations:** 1 Laboratório de Biotecnologia do Câncer, Centro de Desenvolvimento Tecnológico, Biotecnologia, Federal University of Pelotas (UFPel),, Pelotas, Brazil; 2 Laboratório de Pesquisa em Bioquímica e Biologia Molecular de Micro-organismos, Centro de Ciências Químicas, Farmacêuticas e de Alimentos, Federal University of Pelotas (UFPel),, Pelotas, Brazil; 3 Laboratório de Lipidômica e Bio-orgânica, Centro de Ciências Químicas, Farmacêuticas e de Alimentos, Federal University of Pelotas (UFPel), Pelotas, Brazil; 4 Heterocycle Chemistry Nucleus (NUQUIMHE), Federal University of Santa Maria, Santa Maria, Brazil; 5 Faculdade de Medicina (FAMED), Curso de Farmácia, Federal University of Rio Grande (FURG), Rio Grande, Brazil

**Keywords:** cancer cell pharmacology, mitochondrial apoptosis, nano-enabled drug delivery, natural product pharmacology, redox signaling, sesquiterpene-rich essential oil

## Abstract

**Introduction:**

The pharmacological application of essential oils in cancer therapy is frequently limited by poor bioavailability, physicochemical instability, and insufficient cytotoxic potency. Nano-enabled delivery systems have emerged as pharmacological strategies to enhance the therapeutic performance of natural products; however, their pharmacological mechanisms remain incompletely understood.

**Methods:**

We investigated the pharmacological effects of a nanoemulsion incorporating Cyperus scariosus essential oil (NE-CSEO) in human bladder (5637) and lung (A549) cancer cells. Chemical composition was determined by GC-MS, while physicochemical properties were assessed by particle size, polydispersity index, and zeta potential. In vitro cytotoxic effects were evaluated by cell viability assays, and transcriptional responses were explored through gene expression analysis of apoptosis-, antioxidant-, and cell cycle-related genes.

**Results:**

NE-CSEO formed a nanoscale emulsion (115 nm) and produced a concentration-dependent reduction in cell viability after 24 h of treatment, with measurable IC50 values of 4.99 ± 0.22 µg/mL and 2.73 ± 0.54 µg/mL in 5637 and A549 cells, respectively, whereas CSEO and NE-B did not reach 50% inhibition within the concentration ranges tested. At the transcriptional level, NE-CSEO increased BAX, CASP3, and CASP9 mRNA expression and decreased BCL2 mRNA expression in both cancer cell models. In bladder cancer cells, NE-CSEO additionally increased p21 mRNA expression and modulated CAT and SOD transcripts.

**Discussion:**

These findings indicate that the enhanced in vitro growth-inhibitory activity of NE-CSEO is associated with transcriptional modulation of apoptosis-, antioxidant-, and cell cycle-related genes. However, direct functional confirmation of apoptosis, including protein-level apoptotic signaling and caspase activation, requires further investigation. NE-CSEO provides a basis for further preclinical investigation of nano-enabled natural compounds in cancer models.

## 1 Introduction

Cancer remains a major global health challenge and continues to rank among the leading causes of morbidity and mortality worldwide. Current projections indicate that annual cancer diagnoses may exceed 28 million cases by 2040, representing an increase of more than 50% compared with 2020, while cancer-related deaths could reach 16.3 million per year in the absence of effective prevention, early detection, and improved therapeutic strategies (1). Despite advances in conventional treatments, therapeutic resistance, limited selectivity, and severe adverse effects remain critical obstacles, underscoring the urgent need for innovative anticancer approaches with improved efficacy and, ultimately, the potential for improved selectivity (2, 3).

Among malignant diseases, lung cancer remains the most frequently diagnosed tumor type worldwide and is associated with the highest mortality rate (1). Bladder cancer, although less prevalent, represents a substantial clinical and economic burden due to its high recurrence rates, which may reach 30–54% depending on tumor grade, necessitating repeated therapeutic interventions and long-term monitoring (4, 5). Together, these malignancies exemplify the limitations of current oncological therapies and highlight the importance of developing novel treatment strategies capable of improving therapeutic efficacy while minimizing systemic toxicity.

In recent years, natural products have re-emerged as valuable sources of bioactive molecules for cancer therapy, offering structural diversity and multimodal biological activity. In particular, essential oils have attracted increasing attention due to their high content of lipophilic secondary metabolites, broad pharmacological spectrum, and capacity to modulate key cellular pathways involved in tumor progression and cell death (6–8). However, the therapeutic application of essential oils is frequently hindered by poor aqueous solubility, volatility, chemical instability, and limited bioavailability, which restrict their clinical potential.

To address these limitations, nanotechnology-based delivery systems have been increasingly explored as platforms to enhance the pharmacological performance of natural compounds. Nanoemulsions, in particular, have been reported to improve the physicochemical handling and retention of volatile components, enhance solubility under physiological conditions, facilitate cellular delivery, and modulate drug-release profiles (9–11). Numerous studies have demonstrated that nanoencapsulation of essential oils significantly potentiates their anticancer activity, enabling more efficient targeting of tumor cells and amplification of cytotoxic effects compared with non-formulated oils (6).

*Cyperus scariosus*, commonly known as Nagarmotha, is an aromatic perennial plant widely used in traditional medicine and the fragrance industry. Its rhizomes are rich in essential oils predominantly composed of sesquiterpenes, a class of compounds known for diverse biological activities, including anti-inflammatory, antimicrobial, and antitumor effects (12–15). Previous chemical analyses have consistently identified sesquiterpenes as the major constituents of *Cyperus* species essential oils, suggesting their central role in the observed pharmacological properties (15, 16). Importantly, sesquiterpenes have been reported to modulate apoptosis, oxidative stress, and tumor-associated signaling pathways, positioning them as promising candidates for anticancer drug development (6, 17).

Despite the recognized biological potential of *C. scariosus* essential oil, its antitumor efficacy in conventional formulations remains limited, and its mechanistic effects in cancer cells are not fully understood. Incorporation into nanostructured delivery systems represents a rational strategy to improve the physicochemical and biological performance of essential oils. The 5637 and A549 cell lines were selected as complementary *in vitro* models representing two distinct epithelial malignancies, namely bladder and lung cancer, respectively. In addition to their different tissue origins, these cell lines exhibit distinct molecular characteristics that are relevant to the pathways investigated in the present study. The 5637 bladder cancer cell line carries mutations in *TP53* and has been widely used as a model to investigate cell-cycle regulation, apoptosis, and responses to anticancer agents (18). In contrast, A549 lung cancer cells harbor alterations in the KEAP1–NRF2 pathway, resulting in constitutive NRF2 activity and enhanced expression of antioxidant and cytoprotective genes (19). The use of these two models therefore allowed us to evaluate whether the effects of NE-CSEO were consistent across different tumor types while also identifying cell type-dependent responses related to apoptosis, antioxidant defense, and cell-cycle regulation.

Therefore, the present study aimed to develop and characterize a nanoemulsion incorporating *C. scariosus* essential oil and to evaluate its effects on cell viability in human bladder (5637) and lung (A549) cancer cell lines. Furthermore, we sought to investigate transcriptional responses associated with apoptosis, antioxidant defense, and cell cycle-related pathways following NE-CSEO treatment. By integrating natural product pharmacology with nanotechnology-based delivery, this work provides an *in vitro* characterization of the cytotoxic and transcriptional responses to the development of innovative therapeutic strategies for cancer treatment.

## 2 Materials and methods

### 2.1 Acquisition and chemical characterization of *Cyperus scariosus* essential oil

CSEO was commercially obtained from Ferquima (São Paulo, Brazil) following steam distillation of the roots of mature plants. The composition of CSEO was identified at the Laboratory of Innovation and Solutions in Chemistry (INNOVASCHEM), located at the Capão do Leão Campus, Federal University of Pelotas. For this analysis, 10 μL of pure CSEO was solubilized in hexane (Merck, Germany) and analyzed using Gas Chromatography-Mass Spectrometry (GC-MS), Shimadzu^®^ model GC-MS-QP 2010SE, equipped with an AOC-20i auto-injector. The conditions were as follows: RTx-5MS capillary column (30 m × 0.25 mm × 0.25 μm); carrier gas flow rate: 1.20 mL.min-1; carrier gas: Helium; column temperature was initially set at 100 °C (1 min) and gradually increased to 300 °C (4 min) at a rate of 1 °C/min; injector temperature was 260 °C. Mass spectra were recorded in the range of 35–700 m/z. The major constituents of the oil were identified based on their retention times in the GC and by comparing the mass spectra with those in the equipment’s library.

### 2.2 Preparation of nanoemulsion

Nanoemulsions were prepared according to the method described by Giongo et al. (2016) (20), with modifications, using the phase inversion technique. The nanoemulsion containing *Cyperus scariosus* essential oil (NE-CSEO) was obtained by injecting the oil phase (2% *C. scariosus* essential oil and 2% sorbitan monooleate) into the aqueous phase (2% polysorbate 80 and ultrapure water) under high agitation using a T18 Ultra-Turrax (Ika) at 10,000 rpm. Subsequently, the stirring speed was increased to 17,000 rpm, and after approximately 10 minutes, it was reduced to 15,000 rpm, maintaining agitation at this speed for the same duration. For comparison, a control formulation (NE-B) was prepared using a caprylic/capric triglyceride (MCT) mixture instead of *C. scariosus* essential oil. All formulations were prepared and stored protected from light and under refrigeration.

#### 2.2.1 Physicochemical characterization of nanoemulsion

The mean particle diameter, polydispersity index (PDI), and zeta potential of the samples were evaluated using a Zetasizer Lab (Zetasizer Lab, Malvern Instruments). Measurements were performed using mixed-mode light scattering with phase analysis (M3-PALS). The samples were thermostated at 25 °C and measurements were taken after the emulsions were diluted 200-fold in ultrapure water. The analyses were performed in triplicate (n = 3). The results are expressed as the mean of the measurements performed ± standard deviation. The experiments were carried out at the Center for Organic Analysis and Research (NAPO) of the Federal University of Santa Maria.

### 2.3 *In vitro* assessment of cytotoxicity

#### 2.3.1 Cell culture

This study was performed using cancer cells 5637 (bladder) and A549 (lung), all obtained from Rio de Janeiro Cell Bank (PABCAM, Federal University of Rio de Janeiro, Brazil) and were used as received from the cell bank. The cell lines were supplied with quality-control documentation, including certification of absence of mycoplasma contamination based on testing performed by the cell bank. No additional cell line authentication was performed in our laboratory. The 5637 cell line was cultured in RPMI-1640 medium, while A549 cell in DMEM medium. All culture media were supplemented with fetal bovine serum in a final concentration of 10%, 1% of Penicillin/Streptomycin and 1% of Amphotericin B. The cultivation conditions were also the same for all lineages, being 37 °C and 5% of CO_2_ in a humidified atmosphere incubator.

#### 2.3.2 Cell viability assay

Cell viability was assessed via the MTT cytotoxic assay using the cell lines: 5637 and A549 (2 × 10^4^ viable cells per well), which were incubated in the wells of a 96-well plate containing the appropriate medium. The plates were then cultured for 24 h at 37 ˚C under the conditions of 5% CO_2_ and 95% humidity, followed by treatment of cells with different concentrations (10, 50, 100, 150, 200, 250 and 300 μg/mL) of the pure essential oil from *Cyperus scariosus* (CSEO). The nanoemulsion containing the essential oil of *C. scariosus* (NE-CSEO) was evaluated at the concentrations 2, 4, 6, 8, 10 and 50 μg/mL. The blank nanoemulsion, containing all components of the formulation, except for *C. scariosus* essential oil (NE-B), was used in the same volumes as the complete nanoemulsion. The exposure time was 24 hours. A negative control, containing untreated cells, was used in the experiment. After incubation, the medium was removed and 100 μL of MTT solution (3-(4,5-dimethylthiazol-2-yl)-2,5-diphenyltetrazolium bromide) (5 µg/mL) was added to each well, followed by 3 h of incubation. Afterward, the medium was removed again and 100 μL of DMSO was added to each well to solubilize the formazan crystals. The reduction of MTT to formazan, which is in direct proportion to the number of living cells, was examined in a microplate reader (Thermo Plate TP-Reader) at 492 nm. The results were expressed as the percentage of viable cells relative to the untreated cells. All observations were validated in a minimum of three independent experiments. All experiments were performed in triplicate. The percent inhibition of cellular growth was determined as follows: inhibitory rate = (1 - Abs_492treated cells_/Abs_492control cells_) × 100. The IC_50_ value (50% inhibition of cell growth) was calculated using GraphPad Prism 8.0. All data were expressed as mean ± SEM, p values < 0.05 were considered statistically significant.

#### 2.3.3 Gene expression

To analyze the gene expression, 5637 and A549 cells were seeded (5 × 10^5^ cells per well) in 6-well plates and incubated during 24 h with IC_50_ concentration of NE-CSEO. Afterwards, the cells were collected, and the total mRNA was extracted from the cell using TRIzol reagent (Invitrogen™, Carlsbad, USA) and quantified by Nanovue Plus Spectrophotometer™ (GE^®^). The cDNA synthesis was performed using HighCapacity cDNA Reverse Transcription kit (Applied Biosystems™, UK) according to the manufacturer’s protocol. Real time PCR reactions were run on a Stratagene Mx3005P Real-Time PCR System (Agilent Technologies, Santa Clara, CA, USA) using SYBR Green PCR Master Mix (Applied Biosystems, UK). The primers used were human *p21*, *p53*, *CAT*, *SOD*, *BAX*, *BCL2*, *CASP3* and *CASP9*. Gene expression was normalized using *GAPDH* as the reference gene. The sequences of primers are described in **Table 1**.

**Table 1:** Sequences of the genes (forward and reverse) used for gene expression analysis.

Gene	Sequence 5’-3’
*GAPDH*	F: ACAACTTTGGTATCGTGGAAGG R: GCCATCACGCCACAGTTTC
*p21*	F: TGTCCGTCAGAACCCATGC R: AAAGTCGAAGTTCCATCGCTC
*p53*	F: AGCGAGCACTGCCCAACA R: CACGCCCACGGATCTGAA
*CAT*	F: TTTCCCAGGAAGATCCTGAC R: ACCTTGGTGAGATCGAATGG
*SOD*	F: GGAAGCCATCAAACGTGACT R: CTGATTTGGACAAGCAGCAA
*BAX*	F: ATGCGTCCACCAAGAAGC R: ACGGCGGCAATCATCCTC
*BCL2*	F: GGTGGGGTCATGTGTGTGG R: CGGTTCAGGTACTCAGTCATCC
*CASP3*	F: CAGTGGAGGCCGACTTCTTG R: TGGCACAAAGCGACTGGAT
*CASP9*	F: CCAGAGATTCGCAAACCAGAGG R: GAGCACCGACATCACCAAATCC

CAT, Catalase enzyme; SOD, Superoxide dismutase; CASP3, Caspase-3; Casp9, Caspase-9; Bax, BCL2-Associated X protein; BCL2, BCL2 Apoptosis Regulator; p21, protein 21; p53, tumor protein 53.

### 2.4 Statistical analysis

The data are presented as the mean ± standard error of the mean (SEM). Evaluation of the results regarding the MTT was performed using the two-way analysis of variance (ANOVA), followed by Bonferroni’s *post hoc* test. IC50 values, when estimable within the tested concentration range, were independently calculated for each biological replicate using nonlinear regression, and results are presented as mean ± SEM of three independent experiments. No between-group statistical comparison of IC50 values was performed when an IC50 could not be determined. The quantitative Real-Time PCR, were performed using the unpaired t-test. For all tests, a statistical program (GraphPad Prism 8 Software Inc., San Diego, CA, USA) was used. The limit of statistical significance was set at p < 0.05.

## 3 Results

### 3.1 Chemical characterization of CSEO and physicochemical characterization of NE-CSEO

GC-MS analysis of CSEO was performed to characterize the chemical composition of the EO. Seventeen compounds were detected, collectively representing 100% of the total composition of the oil used in this study (**Figure 1**, **Table 2**). The major constituents identified were cyperene (37.13%), followed by cyperotundone (15.05% and 5.11%) and rotundene (10.65%).

**Figure 1:**
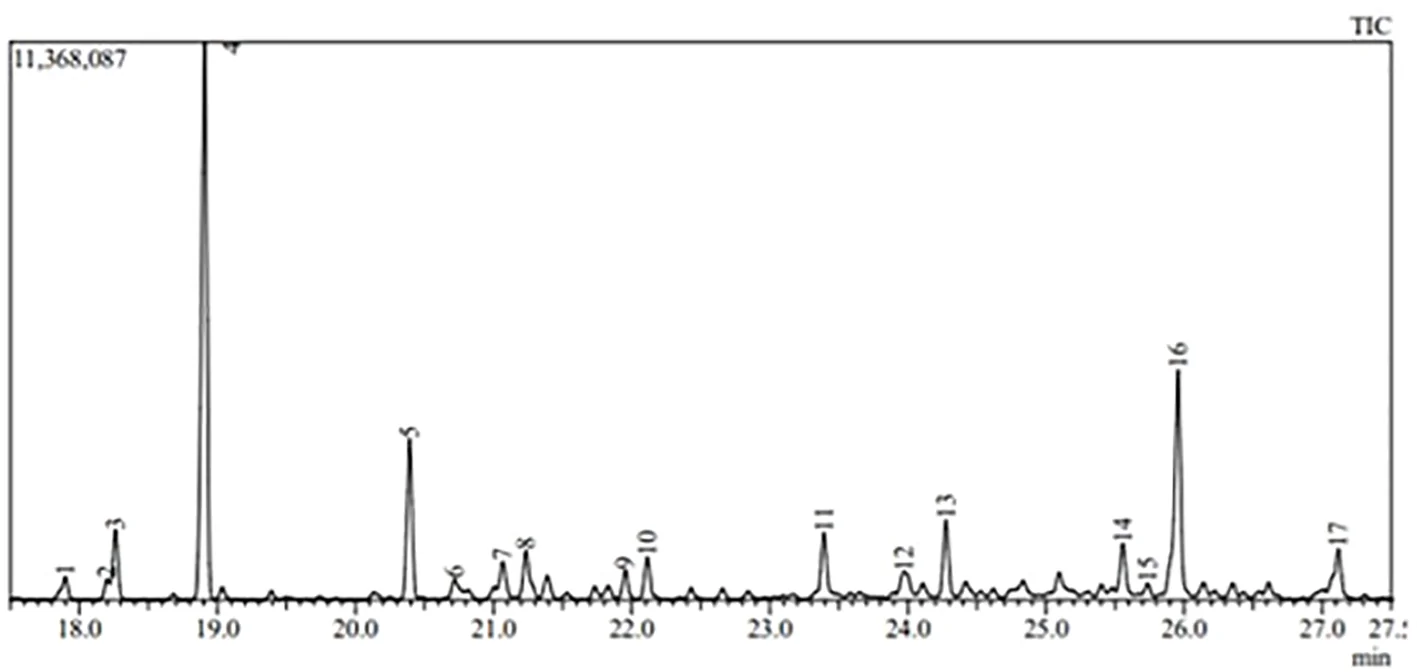
Chromatogram profile obtained by GC-MS of the *Cyperus scariosus* essential oil. Numbered peaks correspond to the identified constituents.

**Table 2:** Chemical composition of *Cyperus scariosus* essential oil.

Compound name	Retention time	Area (%)
Cyperene	18.906	37.13
Cyperotundone	25.956	15.05
Rotundene	20.391	10.65
Cyperotundone	24.275	5.11
Copaene	18.262	4.65
Caryophyllene oxide	23.391	3.99
Mustakone	25.555	3.55
Valencene	21.233	3.21
α-cyperone	27.118	3.09
Cyperene epoxide	22.111	2.69
7-Isopropenyl-1-methyl-4-methylenedecahydroazulene	21.066	2.42
Cadina-1(6),4-diene	21.953	1.88
Cyepero-2,4-diene	17.898	1.51
15-hidroxi- α -muurolene	23.974	1.51
β-Gurjunene	18.200	1.38
γ-Gurjunene	20.717	1.29
2-naphthalenemethanol, 3,4,6,7,8,8a-hexahydro-5-methyl-8-(1-methylethyl)-,(8R-cis)	25.736	0.90

Physicochemical characterization of nanoemulsions revealed notable differences in particle size, polydispersity index (PDI) and surface charge (**Table 3**). The nanoemulsion containing the crude essential oil (NE-CSEO) exhibited a mean particle diameter of 115 ± 9.93 nm, a PDI of 0.307 ± 0.0329 and a zeta potential of -34.61 ± 1.78 mV. In comparison, the NE-B group displayed a smaller particle size of 80 ± 0.38 nm, a PDI of 0.408 ± 0.010 and a more negative surface charge (-38.02 ± 1.00 mV).

**Table 3:** Physicochemical properties of nanoemulsion containing CSEO (NE-CSEO) and blank nanoemulsion (NE-B).

Nanoemulsion	Particle Diameter (nm)	Polydispersity Index	Zeta Potential (mV)
NE-CSEO	115 ± 9.928	0.307 ± 0.0329	-34.61 ± 1.782
NE-B	80 ± 0.378	0.408 ± 0.010	-38.02 ± 1.003

Data are presented as mean of three measurements ± standard deviation.

### 3.2 Effects of CSEO and NE-CSEO on cell viability

The effects of *C. scariosus* essential oil (CSEO), its nanoemulsion (NE-CSEO) and blank nanoemulsion (NE-B) were evaluated in bladder (5637) and lung (A549) cancer cell lines after 24 hours of treatment. The MTT assay showed that NE-CSEO produced a greater concentration-dependent reduction in cell viability than non-nanoemulsified CSEO under the experimental conditions evaluated. In cell line 5637, NE-CSEO demonstrated a more pronounced reduction in cell viability, with a progressive reduction in cell viability as concentrations increased (inhibition ranging from 1.14 to 84.34%). The groups treated with CSEO (38.25% of inhibition at the highest concentration 300 μg/mL) and NE-B (36.5% of inhibition at the highest concentration 50 μg/mL, on the other hand, showed no significant effect at the concentrations tested (**Figure 2**).

**Figure 2:**
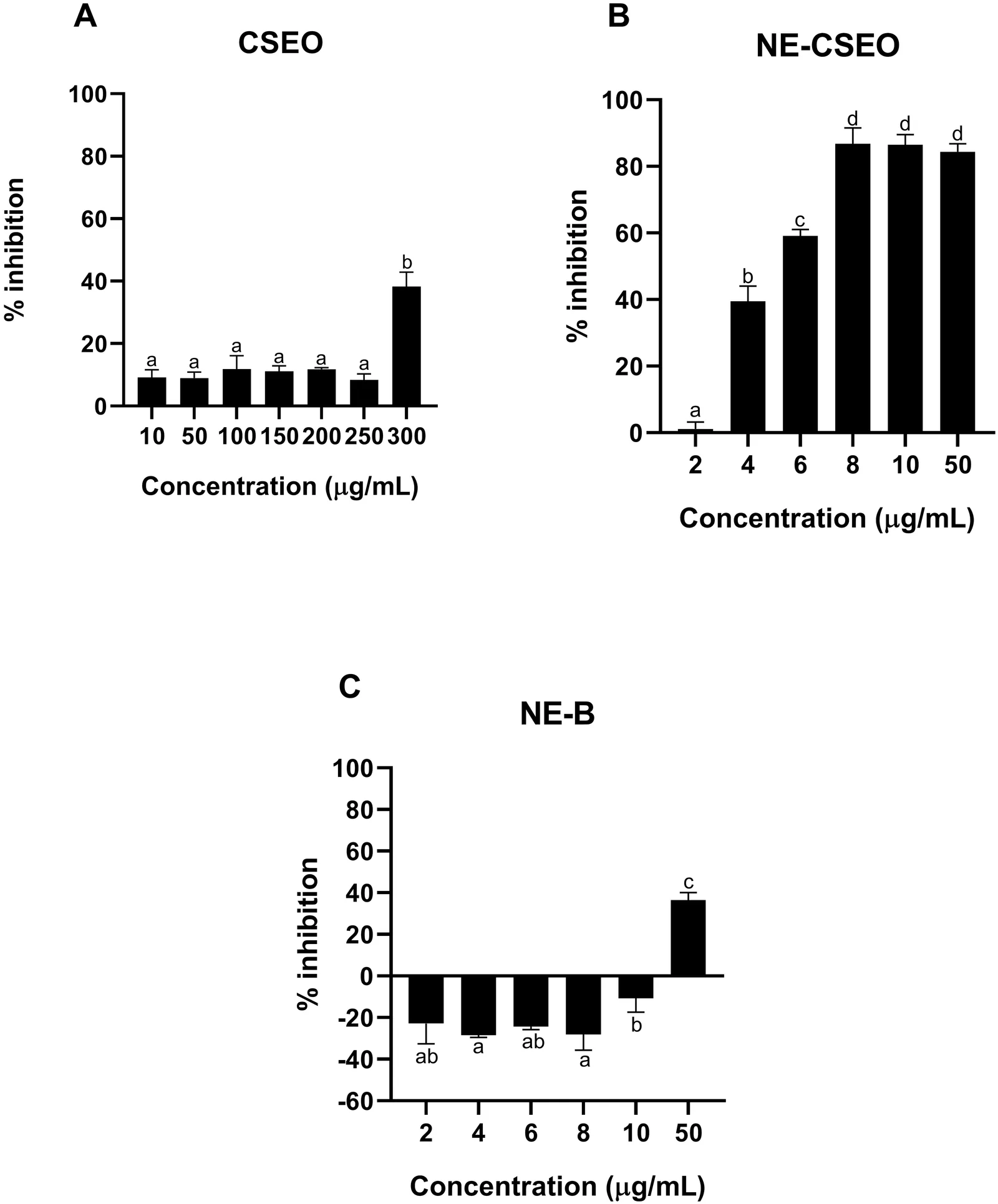
Effect of *Cyperus scariosus* essential oil (CSEO), CSEO nanoemulsion (NE-CSEO), and blank nanoemulsion (NE-B) on the viability of 5637 bladder cancer cells. **(A)** CSEO (10–300 μg/mL); **(B)** NE-CSEO (2–50 μg/mL); and **(C)** NE-B (2–50 μg/mL). Cells were exposed for 24 h and viability was determined by MTT assay. Data are presented as mean ± SEM from three independent experiments performed in triplicate. Different letters indicate statistically significant differences among concentrations (p < 0.05).

Similarly, in the A549 cell line, NE-CSEO demonstrated a more pronounced reduction in cell viability, with a progressive reduction in cell viability as concentrations increased (inhibition ranging from 18.04 to 89.55%). The groups treated with pure CSEO (29.69% of inhibition at the highest concentration 300 μg/mL) and NE-B (38.16% of inhibition at the highest concentration 50 μg/mL, on the other hand, showed no significant effect at the concentrations tested (**Figure 3**). These results suggest that nanoemulsification increased the growth-inhibitory effect of the essential oil under the tested conditions.

**Figure 3:**
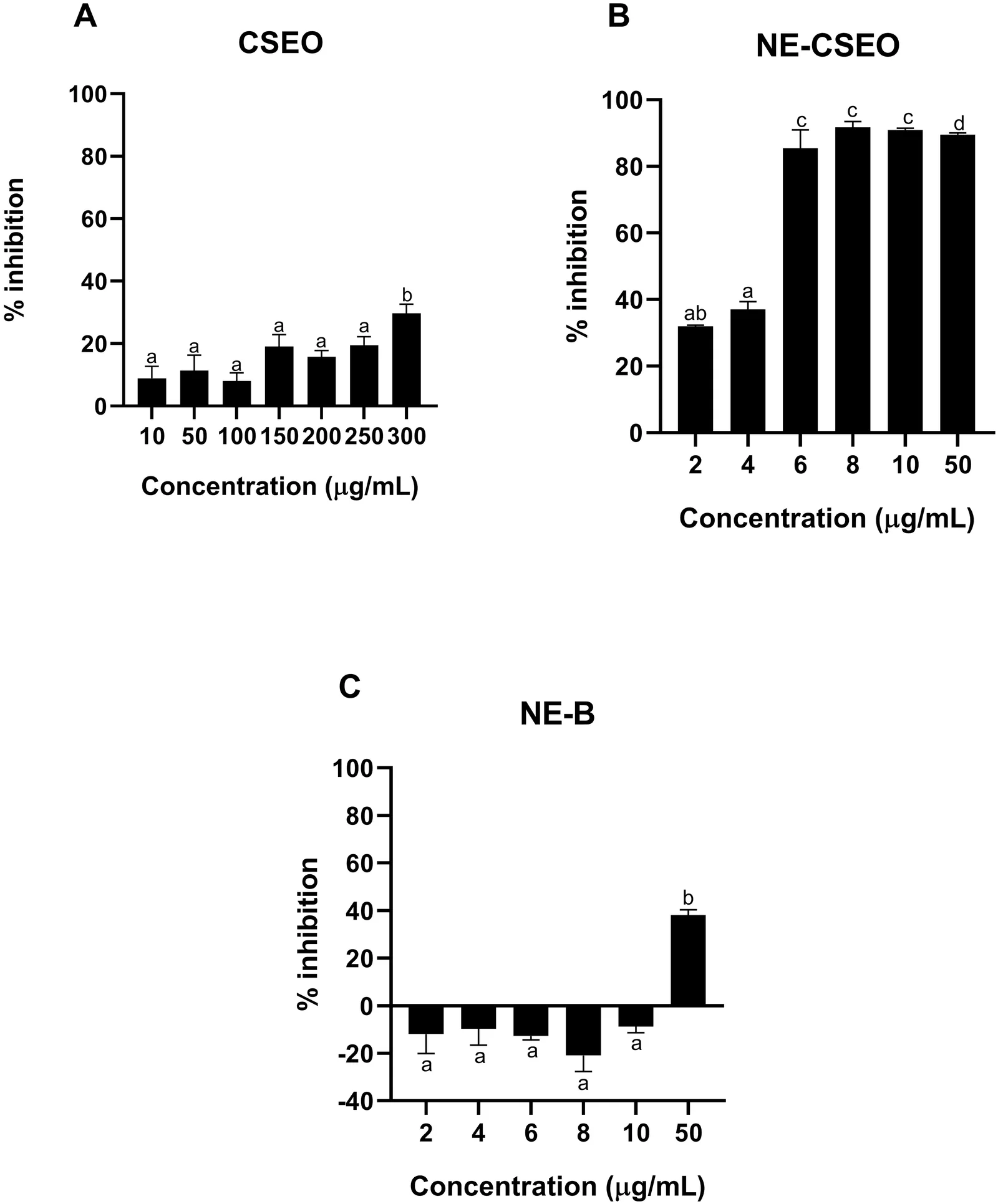
Effect of *Cyperus scariosus* essential oil (CSEO), CSEO nanoemulsion (NE-CSEO), and blank nanoemulsion (NE-B) on the viability of A549 lung cancer cells. **(A)** CSEO (10–300 μg/mL); **(B)** NE-CSEO (2–50 μg/mL); and **(C)** NE-B (2–50 μg/mL). Cells were exposed for 24 h and viability was determined by MTT assay. Data are presented as mean ± SEM from three independent experiments performed in triplicate. Different letters indicate statistically significant differences among concentrations (p < 0.05).

IC_50_ values were assessed after 24 h of treatment in 5637 (bladder cancer) and A549 (lung cancer) cells. NE-CSEO was the only treatment for which an IC_50_ could be determined within the concentration ranges tested, with values of 4.99 ± 0.22 μg/mL and 2.73 ± 0.54 μg/mL in 5637 and A549 cells, respectively. CSEO and NE-B did not reach 50% inhibition of cell viability within the evaluated concentration ranges; therefore, IC_50_ values could not be calculated for these groups and no statistical comparison among IC_50_ values was performed (**Table 4**). The NE-B group did not significantly reduce cell viability under the tested conditions, supporting the interpretation that the growth-inhibitory effect observed with NE-CSEO is associated with the presence of *C. scariosus* essential oil. These results highlight the effectiveness of the nanoemulsion in potentiating the growth-inhibitory activity of the essential oil under the experimental conditions evaluated.

**Table 4:** IC_50_ (median inhibitory concentration) values ​​of *Cyperus scariosus* essential oil (CSEO), nanoemulsion with CSEO (NE-CSEO) and blank nanoemulsion (NE-B) on bladder (5637) and lung (A549) cancer cell lines after 24 h of treatment.

Cell line	IC_50_ (μg/mL)		
	CSEO	NE-CSEO	NE-B
5637	nd	4.99 ± 0.22	nd
A549	nd	2.73 ± 0.54	nd

nd, not detected (50% inhibition of cell viability was not reached at the concentrations tested). No statistical comparison among IC_50_ values was performed because IC_50_ could not be determined for CSEO and NE-B.

Data are expressed as mean ± standard error of the mean (SEM) of three independent experiments.

### 3.3 Effects of NE-CSEO on apoptosis-, antioxidant, and cell cycle-related gene expression

As shown in **Figure 4A**, we can see that bladder carcinoma cells when exposed to treatment with NE-CSEO had an increase in *CAT* mRNA expression (p < 0.001), when compared to the control. The same was observed in **Figure 4B**, which showed an increase in the relative expression of the antioxidant enzyme *SOD* mRNA expression (p < 0.05) in cells treated with NE-CSEO, when compared to the control in bladder carcinoma cells. In the same sense, in **Figures 4C and D** we can see that treatment with NE-CSEO in bladder cancer cells led to an increase in the relative expression of the *CASP3* mRNA expression (p < 0.01) and *CASP9* mRNA expression (p < 0.001) genes respectively when compared to the control.

**Figure 4:**
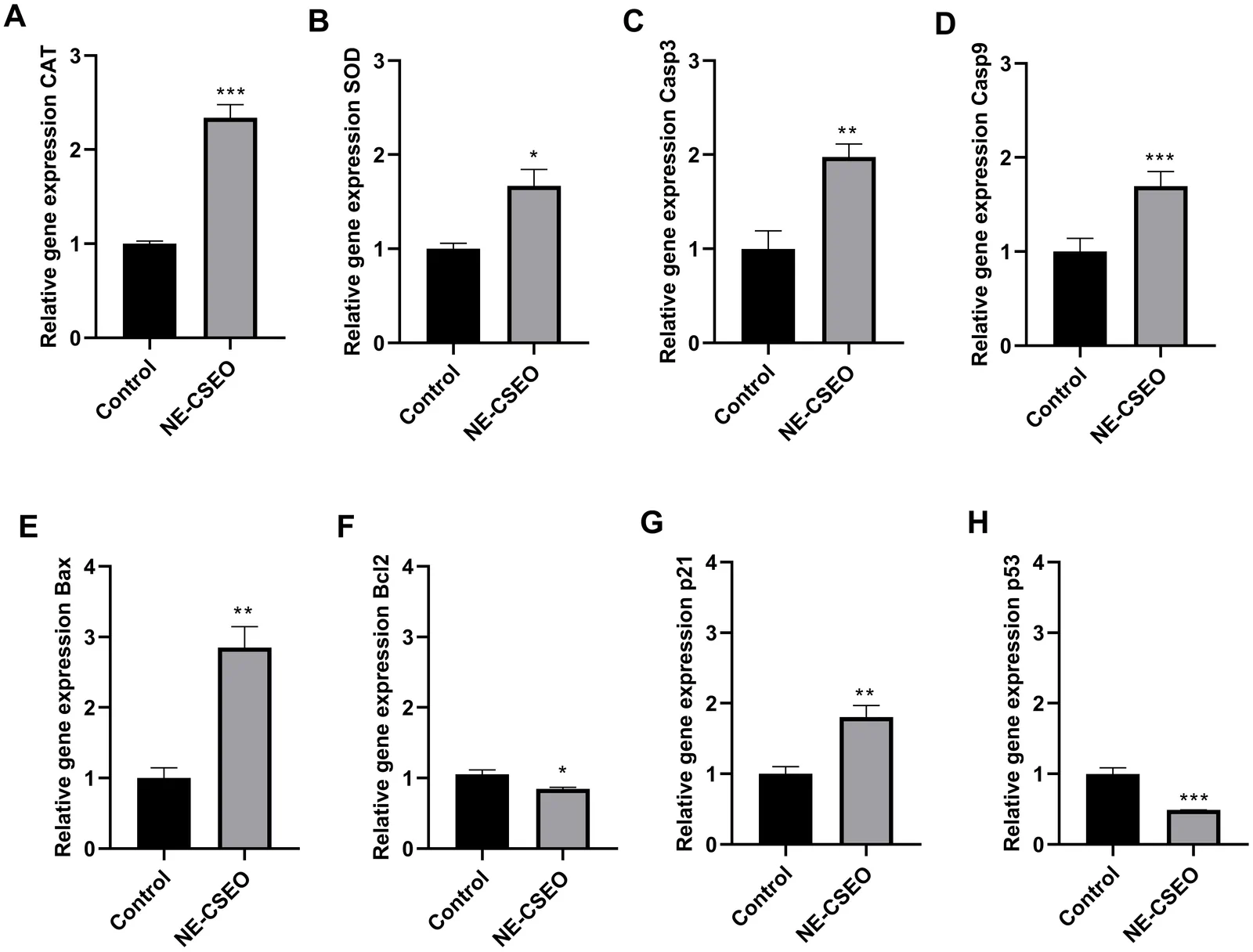
NE-CSEO modulation of apoptosis-, antioxidant-, and cell cycle-related gene expression in 5637 bladder cancer cells. Relative mRNA expression levels of **(A)** catalase (CAT), **(B)** superoxide dismutase (SOD), **(C)** caspase-3 (*CASP3*), **(D)** caspase-9 (*CASP9*), **(E)** *BAX*, **(F)** *BCL2*, **(G)** *p21*, and **(H)** *p53* were evaluated by RT-qPCR after 24 h of treatment with NE-CSEO at 4.99 μg/mL. Gene expression was normalized to *GAPDH* and expressed relative to untreated control cells. Data are presented as mean ± SEM. Statistical significance was determined using the T test (*p < 0.05, **p < 0.01, ***p < 0.001).

In **Figure 4E** we can see that there was an increase in the relative expression of the *BAX* mRNA expression (p < 0.01) in bladder cancer cells that received treatment with NE-CSEO compared to the control. On the other hand, in **Figure 4F**, we can see that there was a decrease in the relative expression of the *BCL2* mRNA expression in cells that were treated with NE-CSEO when compared to the control (p < 0.05). Moreover, in **Figure 4G**, we can see that there was an increase in the expression of the p21 mRNA expression (p < 0.01) in the bladder cancer cells that received the NE-CSEO when compared to the control. When the cells were treated with the NE-CSEO, they had a decrease in the relative gene expression of the *p53* mRNA expression (p < 0.001) when compared to the control (**Figure 4H**). This transcriptional profile is consistent with modulation of genes associated with the intrinsic apoptotic pathway.

In **Figure 5A**, we can observe the increase in the relative expression of the *BAX* mRNA expression (p < 0.01) in lung cancer cells that received treatment with NE-CSEO compared to the control. On the other hand, in **Figure 5B**, we can see that there was a decrease in the relative expression of the *BCL2* mRNA expression in cells that were treated with NE-CSEO when compared to the control (p < 0.001). In the same sense, the **Figures 5C** and **D** when the cells received the treatment with NE-CSEO in lung cancer cells led to an increase in the relative expression of the *CASP3* mRNA expression (p < 0.05) and *CASP9* mRNA expression (p < 0.001) genes, respectively, when compared to the control.

**Figure 5:**
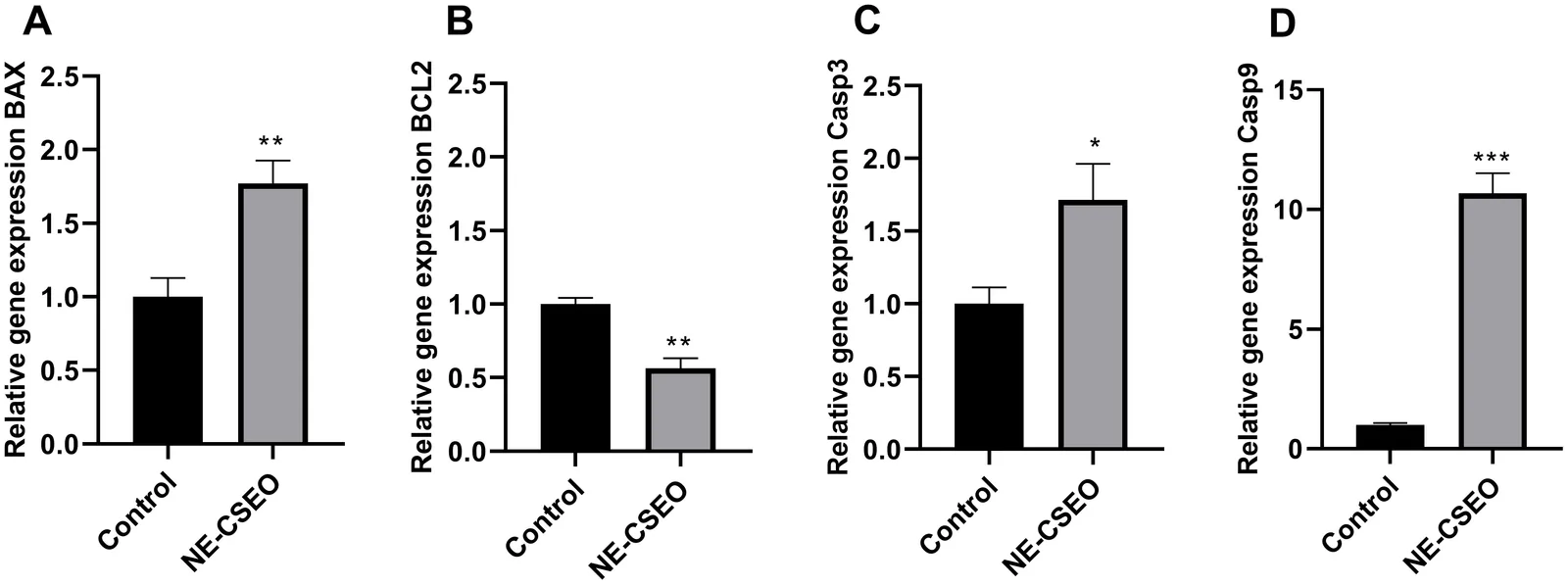
NE-CSEO modulation of apoptosis-related gene expression in A549 lung cancer cells. Relative mRNA expression levels of **(A)** *BAX*, **(B)** *BCL2*, **(C)** caspase-3 (*CASP3*), and **(D)** caspase-9 (*CASP9*) were evaluated by RT-qPCR after 24 h of treatment with NE-CSEO at 2.73 μg/mL. Gene expression was normalized to GAPDH and expressed relative to untreated control cells. Data are presented as mean ± SEM. Statistical significance was determined using the T test (*p < 0.05, **p < 0.01, ***p < 0.001).

## 4 Discussion

Plants from the *Cyperus* genus have been extensively investigated due to their broad spectrum of biological activities, including antitumor effects (15). *Cyperus scariosus* has long been used in traditional medicine and is recognized for multiple pharmacological properties, such as antihypertensive, antispasmodic, anticonvulsant, and antimicrobial activities (21). Among plant-derived bioactive agents, essential oils represent an attractive therapeutic source owing to their high concentration of lipophilic secondary metabolites, enhanced membrane permeability, and multimodal biological activity (7, 8). However, their pharmacological application in oncology is frequently limited by poor aqueous solubility, volatility, physicochemical instability, and reduced bioavailability.

To overcome these limitations, nanostructured delivery systems such as nanoemulsions have emerged as effective strategies to enhance the pharmacological performance of essential oils (11). In this context, the present findings indicate that nanoformulation functions as a pharmacological potentiation strategy, enhancing the *in vitro* growth-inhibitory activity of *C. scariosus* essential oil. Rather than representing a formulation-centered phenomenon, the enhanced cytotoxic response observed with NE-CSEO was accompanied by transcriptional modulation of genes associated with pathways involved in cancer cell survival and death. In both bladder and lung cancer models, NE-CSEO modulated molecular markers associated with intrinsic apoptotic signaling, as evidenced by *BAX* upregulation, *BCL2* downregulation, and increased expression of caspases-3 and -9, resulting in a transcriptional profile consistent with modulation of the intrinsic apoptotic pathway. This pharmacological profile aligns with previous reports describing sesquiterpene-mediated induction of caspase-dependent apoptosis through disruption of mitochondrial homeostasis and redox balance (6, 17, 22, 23). However, the present study evaluated transcript levels only and therefore does not establish whether these transcriptional changes result in functional caspase activation or apoptotic cell death. Based on the reported properties of nano-enabled delivery systems, increased intracellular availability may contribute to the observed response, but this mechanism cannot be inferred from the present experiments (9, 11, 24–27). However, this possibility was not directly evaluated in the present study.

Chemical analysis revealed that the essential oil was predominantly composed of sesquiterpenes, particularly cyperene, cyperotundone, and rotundene, which together accounted for approximately 68% of the total composition. This profile is consistent with previous studies reporting sesquiterpenes as the major phytochemical class in *Cyperus* species (15, 16, 28). Sesquiterpenes are widely recognized for their ability to modulate key pharmacological processes involved in tumor progression, including apoptosis, oxidative stress, and mitochondrial function (6, 17). The physicochemical features of the developed nanoemulsion, such as nanoscale droplet size, narrow size distribution, and high negative zeta potential, are consistent with the formation of a nanoscale colloidal system, which likely contributed to the enhanced cytotoxic response observed (29–33).

Beyond the modulation of apoptosis-related genes, NE-CSEO elicited additional transcriptional responses in bladder cancer cells, including changes in the expression of antioxidant-related genes and cell cycle-associated genes. The increased *CAT* and *SOD* mRNA expression in 5637 cells indicate modulation of antioxidant-related gene expression. Cancer cells typically maintain elevated basal levels of reactive oxygen species while relying on antioxidant systems to avoid lethal oxidative damage. Disruption of this redox balance has been increasingly explored as a pharmacological vulnerability in bladder cancer (34–36). Therefore, the observed changes in *CAT* and *SOD* expression should be interpreted as transcriptional modulation of antioxidant-related genes rather than direct evidence of oxidative stress or redox imbalance.

In addition, NE-CSEO promoted upregulation of p21 in bladder cancer cells, suggesting modulation of cell cycle-related pathways through mechanisms independent of p53 activity. This observation is particularly relevant, as 5637 cells harbor TP53 alterations that compromise canonical p53-dependent checkpoint control (18). The induction of p21 under these conditions is consistent with the possible involvement of alternative regulatory pathways previously described to operate independently of p53, including suppression of CDK–cyclin complexes and MELK-related signaling (37, 38). These findings suggest a cell type–dependent pharmacological profile of NE-CSEO involving transcriptional modulation of genes associated with cell survival and cell cycle regulation.

In contrast, lung cancer cells exhibited a more restricted transcriptional response, characterized predominantly by modulation of apoptosis-related genes, without significant changes in the antioxidant-related genes evaluated in the present study. This differential response may reflect intrinsic molecular features of A549 cells, including previously described constitutive activation of NRF2 signaling due to KEAP1 mutations, which confer enhanced antioxidant capacity and resistance to oxidative stress-induced cytotoxicity (19, 39). Despite this known redox resilience of A549 cells, NE-CSEO modulated the evaluated apoptosis-related transcripts, highlighting the consistent transcriptional modulation of apoptosis-related genes across distinct tumor phenotypes.

Collectively, these findings demonstrate that, under the experimental conditions evaluated, nanoemulsification substantially enhances the *in vitro* growth-inhibitory activity of *C. scariosus* essential oil. Based on the reported properties of nanoemulsion-based delivery systems, enhanced intracellular availability may contribute to this effect. However, cellular uptake was not directly evaluated in the present study. NE-CSEO was associated with transcriptional modulation of apoptosis-related genes in both cancer cell models, while additional antioxidant- and cell cycle-related transcriptional responses were observed in a cell type–dependent manner. This transcriptional profile supports nano-enabled natural products as potential pharmacological strategies in preclinical cancer models.

Several limitations should be considered when interpreting the present findings. The study was performed exclusively in malignant cell lines and did not include non-malignant controls, limiting the assessment of NE-CSEO selectivity toward cancer cells. Apoptosis- and antioxidant-related responses were evaluated only at the mRNA level. Therefore, the observed changes do not provide definitive evidence of apoptosis or altered redox status. Cellular uptake and intracellular internalization were not directly evaluated, leaving enhanced intracellular delivery as a possible but unverified mechanism. Time-dependent responses were also not characterized. Long-term physicochemical stability was not assessed; therefore, the present characterization should not be interpreted as evidence of long-term formulation stability. Finally, as the study was exclusively *in vitro*, the systemic pharmacological behavior, biodistribution, metabolism, tolerability, and therapeutic efficacy of NE-CSEO remain to be established *in vivo*.

In conclusion, the present study demonstrates that, under the experimental conditions evaluated, nanoemulsification enhances the *in vitro* growth-inhibitory activity of *Cyperus scariosus* essential oil in bladder and lung cancer cells. The nanoformulated system enhanced the *in vitro* growth-inhibitory activity of the sesquiterpene-rich bioactive compounds and was associated with a transcriptional profile characterized by altered expression of genes related to intrinsic apoptotic signaling in bladder and lung cancer cells. In addition to the modulation of apoptosis related genes, NE-CSEO exhibited cell type–dependent transcriptional responses, including modulation of antioxidant-related and cell cycle-related gene expression in bladder cancer cells, while showing a predominant modulation of apoptosis-related genes in lung cancer cells.

Importantly, these findings provide transcriptional evidence that nanoemulsification is associated with modulation of genes involved in cell survival and death pathways; they do not demonstrate functional activation of these pathways. Overall, these findings provide an *in vitro* pharmacological and transcriptional framework for further preclinical investigation of NE-CSEO and related nanoformulations. Complementary functional, biochemical, and cellular uptake studies will therefore be important to further characterize the mechanisms underlying the activity of NE-CSEO.

## Data Availability

The original contributions presented in the study are included in the article/supplementary material, further inquiries can be directed to the corresponding author/s.
